# Genomic Analysis of *emm59* Group A *Streptococcus* Invasive Strains, United States

**DOI:** 10.3201/eid1804.111803

**Published:** 2012-04

**Authors:** Nahuel Fittipaldi, Randall J. Olsen, Stephen B. Beres, Chris Van Beneden, James M. Musser

**Affiliations:** The Methodist Hospital Research Institute, Houston, Texas, USA (N. Fittipaldi, R.J. Olsen, S.B. Beres, J.M. Musser);; Centers for Disease Control and Prevention, Atlanta, Georgia, USA (C. Van Beneden)

**Keywords:** group A Streptococcus, GAS, bacteria, invasive disease, genome sequencing, epidemic, United States, Canada, streptococci

## Abstract

Genomic analysis of type *emm59* group A *Streptococcus* invasive strains isolated in the United States discovered higher than anticipated genetic heterogeneity among strains and identified a heretofore unrecognized monoclonal cluster of invasive infections in the San Francisco Bay area. Heightened monitoring for a potential shift in the epidemic behavior of *emm59* group A *Streptococcus* is warranted.

Group A *Streptococcus* (GAS) causes human diseases ranging in severity from uncomplicated pharyngitis to life-threatening necrotizing fasciitis (*1*). GAS strains have traditionally been classified based on a serologic reaction against the M protein, a polymorphic cell surface adhesin and anti-phagocytic factor; GAS strains are currently typed by sequencing the 5′ hypervariable region of the *emm* gene, encoding M protein (*2**–**4*). Studies of large numbers of GAS isolates causing invasive infections and pharyngitis worldwide have shown that type *emm59* GAS strains rarely cause disease (*5**,**6*). However, an increase in the frequency and severity of invasive infections caused by type *emm59* strains (>500 cases since 2006) has been reported recently in Canada (*7*). One of the most striking features of the *emm59* epidemic in Canada was its rapid spread; invasive *emm59* disease was reported in most Canadian provinces and territories in a matter of only a few years (*7*). By using whole-genome sequencing and animal models of invasive disease, we recently discovered that virtually all type *emm59* GAS invasive cases in Canada were caused by a single, recently emerged, hypervirulent *emm59* clone (*8*).

Whole-genome sequence analysis also revealed distinct spatiotemporal patterns of subclone diversification of the epidemic clone in Canada (*8*). Furthermore, we discovered that several geographically clustered cases of type *emm59* GAS invasive infections in south-central Montana in 2010 were caused by a distinct subclone of the *emm59* epidemic clone that disseminated from Canada (*8*). This finding led us to evaluate the hypothesis that the epidemic *emm59* clone disseminated further and caused invasive infections in other regions of the United States.

## The Study

In this study, we sequenced the genomes of all available invasive *emm59* GAS strains (m = 40) collected during 2000–2009 by the Active Bacterial Core surveillance (ABCs), a core component of the Centers for Disease Control and Prevention Emerging Infections Programs network. ABCs, an active, laboratory- and population-based surveillance system operating in 10 geographically disparate sites across the United States, represents a population of ≈32 million persons under surveillance for invasive GAS infections (www.cdc.gov/abcs/methodology/surv-pop.html).

**Figure A1:**
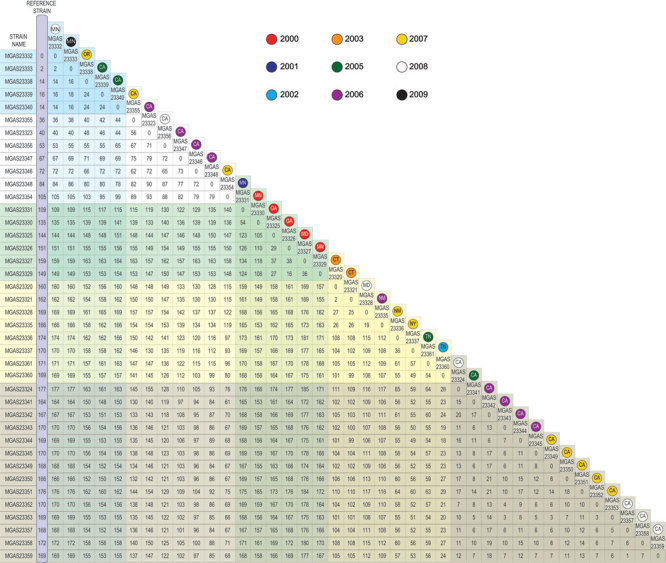
Matrix displaying total number of core genome single-nucleotide polymorphisms separating each individual strain from any other. State and year of isolation are provided for each individual strain. CA, California; CT, Connecticut; GA, Georgia; MD, Maryland; MN, Minnesota; NM, New Mexico; NY, New York; OR, Oregon; TN, Tennessee.

Genome sequencing was performed by using a Genome Analyzer II (Illumina, San Diego, CA, USA) according to the manufacturer’s instructions. Polymorphism discovery and phylogenetic analysis were performed as described (*8*). The reference genome sequence for polymorphism discovery was that of the *emm59* Canadian strain MGAS15252 (GenBank accession no. CP003116). On average, the 40 *emm59* strains in the ABCs sample differed from the reference strain by 157 single-nucleotide polymorphisms (SNPs) and 15 insertions or deletions. We recently reported that, consistent with our hypothesis, we identified 5 strains that were genetically closely related to the epidemic clone in Canada: 1 strain from Oregon, 2 from California, and 2 from Minnesota (*8*). Here, we report that the core genomes (i.e., the ≈1,670-kbp portion of the genome that lacks mobile genetic elements and whose gene content is conserved among all sequenced GAS serotypes) of these 5 strains differed from the core genome of reference strain MGAS15252 by <16 SNPs (Figure A1).

Although our hypothesis regarding further dissemination of this recently emerged clone into the United States was correct, most *emm59* GAS strains collected by the ABCs program were genetically distinct from the clone in Canada. We therefore investigated in more detail the population of *emm59* GAS organisms responsible for invasive disease in the United States. We used the whole-genome SNP data to identify 2 major phylogenetic lineages of *emm59* organisms (Figure, panel A). All strains recovered during 2000 and 2001 (originating from Minnesota, Maryland, and Georgia) form 1 lineage (Figure, panel A, highlighted in green). On average, the core genomes of these strains differed from that of reference strain MGAS15252 by 141 SNPs. The second lineage consists of 22 strains isolated in California, Connecticut, New Mexico, New York, and Tennessee during 2003–2008 (Figure, panel A, highlighted in yellow). On average, the core genomes of these 22 type *emm59* organisms differed from that of reference strain MGAS15252 by 169 SNPs. Seven strains from California, isolated during 2006–2008, were separated from the reference strain by increasing numbers of SNPs (ranging from 36 to 105 SNPs for the most closely to the most distantly related of the 7 strains, respectively) (Figure, panels A).

**Figure:**
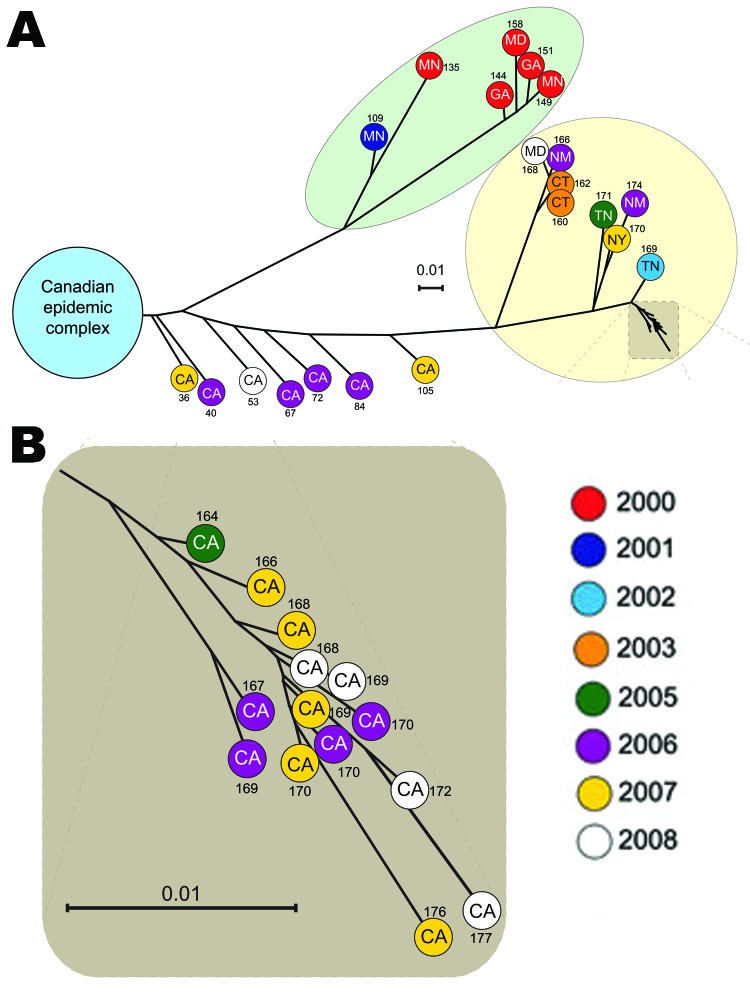
Inferred genetic relationships among 40 *emm59* group A *Streptococcus* (GAS) strains isolated in the United States during 2000–2009, based on 635 concatenated single-nucleotide polymorphism (SNP) loci identified by whole-genome sequencing. A) Phylogenetic tree showing that most of the strains are genetically distinct from an *emm59* GAS clone responsible for >500 cases of invasive disease in Canada. Light green highlight indicates older (US) strains (isolated during 2000–2001) that form 1 clearly differentiated branch of the phylogenetic tree. Yellow highlighting indicates a second lineage isolated in the United States (CA, CT, NM, NY, and TN) during 2003–2008. Circles represent individual isolates. The number of SNPs separating each isolate from strain MGAS15252 from Canada is indicated. Five strains (2 from MN, 2 from CA, and 1 from OR; not represented individually in the phylogenetic tree) are members of the epidemic complex from Canada. B) Magnification of the phylogenetic tree showing a conspicuous discrete clonal complex formed by 14 strains isolated in the San Francisco Bay area during 2005–2008. Genome-wide, these 14 strains are separated by an average of 10 SNPs. CA, California; CT, CT, Connecticut; GA, Georgia; MD, Maryland; MN, Minnesota; NM, New Mexico; NY, New York; TN, Tennessee. A matrix displaying the total number of core genome SNPs separating each individual strain from any other is available in Figure A1.

Closer examination of the second branch of the phylogenetic tree identified a conspicuous group formed by 14 closely related strains isolated from patients in the San Francisco Bay area, California, USA, during 2005–2008 (Figure, panel B). These 14 type *emm59* GAS organisms differed from one another, on average, by only 10 SNPs. The phylogenetic and epidemiologic data suggest that these 14 strains constitute a distinct clone that caused a geographic cluster of invasive infections.

The *emm59* strains causing the epidemic in Canada were isolated in high percentages from patients with bacteremia and soft tissue infections (*7*). In the only other well-documented *emm59* outbreak, which occurred in Scotland (*9*), most of the patients had skin lesions and wound infections (*9*). These data suggest that *emm59* GAS strains have a predilection for abscess formation and soft tissue infection. The most common clinical syndromes associated with infections caused by *emm59* GAS strains in the ABCs collection were similar to those associated with infections caused by all other *emm* types (*10*) and included bacteremia (40%), cellulitis (28%), pneumonia (13%), septic arthritis (5.0%), necrotizing fasciitis (7.5%), and abscess (5.3%). The initial report of type *emm59* GAS strains indicated this *emm* type to be associated with pyoderma and acute glomerulonephritis (*11*). However, the ABCs program tracks only invasive infections, so it would probably not detect most cases of GAS pyoderma and glomerulonephritis.

In Canada, an epidemiologic association with alcohol abuse, homelessness, hepatitis C virus infection, and illicit drug use was identified, suggesting that the *emm59* GAS epidemic was more common among a specific susceptible population consisting primarily of middle-aged persons with underlying medical conditions or histories of substance abuse (*7*). In our study of type *emm59* GAS invasive infection in the United States, we found that 30% of infected persons used illicit drugs and 17.5% abused alcohol; these percentages were higher than those for persons infected with all other *emm* types (*10*). However, substance abuse was higher only among US case-patients with infections caused by strains belonging to the epidemic clone from Canada and among case-patients from the San Francisco Bay area. This finding likely reflects circulation of the *emm59* strain among subpopulations with similar behaviors. Of note, substance abuse has been shown to be a major risk factor for invasive GAS disease in the San Francisco Bay area (*12*).

## Conclusions

Next-generation DNA sequencing technologies are highly successful for infectious disease epidemiology (*13*). The *emm59* GAS organisms causing invasive infections in the United States were closely related and indistinguishable by multilocus sequence typing. The strains could be differentiated from one another only by the use of high-throughput genome sequencing. The level of genetic diversity we identified among *emm59* GAS strains collected by the ABCs program in the United States was considerably greater than that among strains from the epidemic in Canada, where a monoclonal population was found to be responsible for virtually all of the >500 invasive cases reported (*8*). Of note, the high resolving power of whole-genome sequencing also enabled us to discover a cluster of invasive infections caused by very closely related *emm59* GAS strains in the San Francisco Bay area.

Until recently, cases of invasive infection caused by *emm59* GAS have been uncommon in North America. However, the recent epidemic in Canada (*7**,**8*) and the data presented here for the United States suggest a potential shift in the epidemic behavior of *emm59* GAS strains that warrants heightened monitoring awareness by public health authorities.
